# Nondestructive Detection and Early Warning of Pavement Surface Icing Based on Meteorological Information

**DOI:** 10.3390/ma16196539

**Published:** 2023-10-03

**Authors:** Jilu Li, Hua Ma, Wei Shi, Yiqiu Tan, Huining Xu, Bin Zheng, Jie Liu

**Affiliations:** 1 School of Transportation Science and Engineering, Harbin Institute of Technology, Harbin 150090, China; 2 Xingtai Pavement & Bridge Construction Group Co., Ltd., Xingtai 054000, China; 3 Heilongjiang Transportation Investment Group Co., Ltd., Harbin 150000, China

**Keywords:** icy pavement, intelligent monitoring, meteorological parameter, early warning

## Abstract

Monitoring and warning of ice on pavement surfaces are effective means to improve traffic safety in winter. In this study, a high-precision piezoelectric sensor was developed to monitor pavement surface conditions. The effects of the pavement surface temperature, water depth, and wind speed on pavement icing time were investigated. Then, on the basis of these effects, an early warning model of pavement icing was proposed using an artificial neural network. The results showed that the sensor could detect ice or water on the pavement surface. The measurement accuracy and reliability of the sensor were verified under long-term vehicle load, temperature load, and harsh natural environment using test data. Moreover, pavement temperature, water depth, and wind speed had a significant nonlinear effect on the pavement icing time. The effect of the pavement surface temperature on icing conditions was maximal, followed by the effect of the water depth. The effect of the wind speed was moderate. The model with a learning rate of 0.7 and five hidden units had the best prediction effect on pavement icing. The prediction accuracy of the early warning model exceeded 90%, permitting nondestructive and rapid detection of pavement icing based on meteorological information.

## 1. Introduction

Ice refers to a pollutant on a pavement surface formed by rain condensation, melting snow, or humid air in low-temperature environments. Ice adheres to pavement surfaces and masks the surfaces’ texture [1], thereby causing a rapid reduction in pavement anti-sliding characteristics [2]. Accidents on icy pavements have a high occurrence probability and serious consequences. They also gravely threaten the personal safety of drivers [3,4]. The presence of ice on pavements should be accurately detected, and timely warning must be provided to reduce the occurrence of traffic accidents in winter.

The accurate detection of pavement ice may contribute considerably to traffic control decision-making and improve traffic safety [5,6]. Accordingly, various ice sensors with different technologies have been developed to detect pavement conditions. In accordance with the detection method, sensors can be divided into destructive sensing and nondestructive sensing. Destructive detection sensors are installed on pavements to detect ice (e.g., sonic wave [7,8,9], resistance [10], capacitance [11], and vibration [12]). The limitations of the aforementioned sensors, such as sluggish response times and poor durability, prevent their application in pavement engineering. Additionally, nondestructive detection sensors, such as infrared [13,14] and optical fiber [15], are installed above the pavement surface. Although the accuracy of nondestructive sensing technology is high under normal operations, the interference of pavement lights and installation height have potential influence on the technology’s detection accuracy, making stable and accurate detection results difficult to obtain. In addition, the current sensors are relatively expensive. China’s extensive transportation network makes it difficult to use these sensors for coverage applications. Therefore, ice sensors for pavement engineering should be developed considering cost, durability, and accuracy.

Meteorological factors are the main cause of pavement icing. The effects of various factors on the icing state have been studied. Zhang et al. simulated the icing environment to study the nonlinear formation of ice on a cold surface under the action of meteorological factors. The test results showed that the icing time increased with the decrease in temperature and wind speed [16]. Xu et al. measured the icing time of a pavement and established an empirical relationship among pavement temperature, water freezing point, and icing time [17]. Samodurova analyzed the influence of various factors on pavement icing and proposed a linear discriminant forecast function with crucial factors: temperature and precipitation [18,19].

Having clarified the influence of meteorological factors on icing, researchers have also attempted to achieve nondestructive detection and warning of pavement icing using meteorological factors. On the basis of the pavement meteorological data collected by the Pavement Weather Information System, the Swedish Transport Administration studied the effects of pavement temperature and precipitation on the pavement icing state and developed the pavement icing expert analysis system [20,21]. The relevant departments in Germany also attach great importance to pavement weather forecasts. A pavement management department analyzed the influence of temperature and wind speed on pavement icing and proposed a pavement icing warning method that considers the meteorological information in the past 24 h [22]. The National Weather Service in the United States established a pavement information monitoring system. The system can predict the thickness of pavement ice accretion on the basis of the variations in several factors (e.g., air temperature, wind speed, and precipitation rate) [23]. Korotenko investigated the relationship among temperature, water, and pavement icing; a second-order diffusion equation with empirically parameterized flux terms was proposed for the numerical icing forecasting system in Northern Europe and North America [24]. Meanwhile, Martorina and Loglisci conducted thermal mapping of main pavements in Piemonte to infer the pavement temperature along all pavement networks. The researchers proposed a small-scale pavement ice forecasting system in accordance with the variation in air temperature, dew point, wind speed, and cloud amount in the past three hours [25]. Alexander summarized and predicted the icing-sensitive areas of the Danish highway network using observed data (pavement temperature, air temperature, and dew point temperature) from a meteorological station from 2003 to 2007. The researchers suggested improving the quality and accuracy of pavement icing prediction through thermal mapping measurements [26].

Moreover, an accurate, nondestructive detection and early warning model provides essential benefits. Ye et al. showed that the ice early warning system developed by the California Department of Transportation has economized at least USD 1.7 million and reduced the occurrence of accidents by 18% [27]. The models above can predict pavement icing successfully through the nonlinear regression of the historical data of meteorological factors. However, this type of early warning entails some problems. In low-temperature environments, the water on pavements takes some time to completely turn into ice. The abovementioned models are complicated because excessive factors are considered. Too many model input parameters lead to the need for a large calculation capacity and long warning times, which cannot meet the needs of rapid warning of pavement icing. Furthermore, the models exhibit strong temporal and spatial characteristics, which limit their application. With the continuous change in the global climate, the applicability of these models decreases, causing a decline in early warning accuracy. Thus, fast-response, self-adaptive early warning models of pavement icing are needed.

Due to a variety of factors, China’s road management still lacks an effective nondestructive detection and early warning system for pavement icing. In order to eliminate the negative effects of ice on highways in Hebei Province, this paper preliminarily realizes the rapid, nondestructive detection and precise, accurate early warning of pavement icing for the meteorological conditions of Hebei Province. Specifically, this study proposes a pavement surface icing sensor that enables the accurate measurement of icing thickness through the piezoelectric properties of the material. The effects of key meteorological factors on pavement icing were clarified, and an adaptive learning pavement icing warning model was developed to nondestructive detect pavement icing by inputting meteorological parameters. The study provides novel insights into and a technical reference for the safe and efficient operation of pavement infrastructure.

## 2. Sensor and Method

### 2.1. Sensor Development

Accurate acquisition of pavement icing information is the key to improving traffic safety in the winter. Since sensors have a high failure rate and low accuracy when used to obtain pavement icing information because of the hostile natural environment of the pavement, manual observation is still the primary method used to obtain this information today. This observational method relies on the empirical assessment of pavement maintenance workers, which might be very subjective and prone to inaccuracy. In order to increase the precision and automation of ice detection, this paper proposes a durable ice sensor for acquiring icing condition information.

#### 2.1.1. Sensor Design

1. Sensor Principle

**Figure 1:**
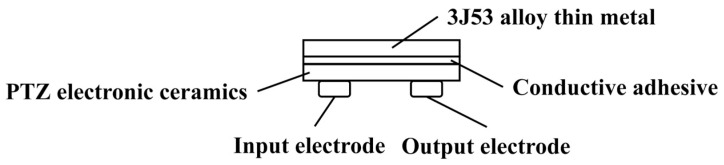
Ice sensing element structure.

Through the change in the overall resonant frequency of the sensing element and the attached material, the sensor determines the thickness of the material on the surface of the sensing element. When the sensor is in operation, a stimulus electrical signal is applied to the input electrode of the sensing element in a certain step, and periodically increases, causing the piezoelectric ceramic to vibrate. The output electrode of the sensor element produces a voltage signal that contains information about the vibration frequency as a result of the inverse piezoelectric effect. The output electrical signal will be greatest when the frequency of the input signal and the resonance frequency of the sensing element coincide. And when the output voltage signal is at its strongest, the frequency of the stimulus signal at the input electrode is the resonant frequency. Figure 1 depicts the structure of the sensing element. The proposed sensor uses PTZ (Piezoelectric Ceramic Transducer) and 3J53 alloy bonded with conductive adhesive as its sensing element.

The sensor measures the resonance frequency of the ice sensing element and the material on its surface to detect the presence of ice or water [28]. The sensor has a measuring range of 0~4 mm. The resonance frequency *f* of the electronic ceramic material is related to its equivalent stiffness and equivalent mass and is calculated as Equation (1). When ice or water are present on the surface of the sensing element, the ice or water form a new whole with the sensing element and change the original equivalent stiffness and equivalent mass of the sensing element. Different amounts of substances have different effects on the equivalent stiffness and equivalent mass of the sensing element, and a regular change in resonant frequency is produced, thus achieving the accurate detection of ice or water thickness.

(1) $$f=\frac{1}{2\pi }\sqrt{\frac{K}{m}}$$

where *K* denotes the equivalent stiffness of the electronic ceramic material, *m* is the equivalent mass of the electronic ceramic material.

**Figure 2:**
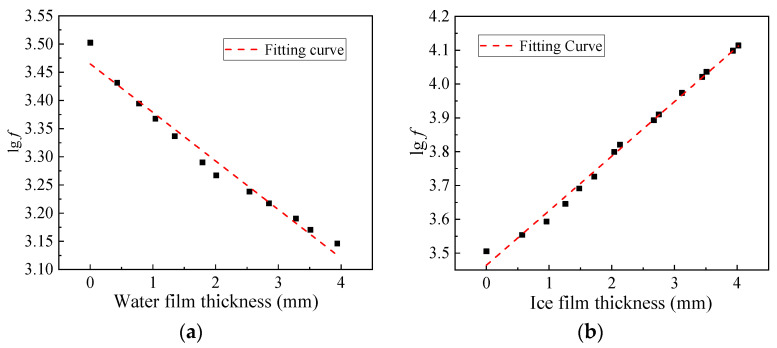
Fitting curve between resonant frequency and film thickness: (**a**) lg*f* and water film thickness; (**b**) lg*f* and ice film thickness.

To monitor the ice thickness on a pavement surface using a sensor, the relationship between the sensor’s resonant frequency and the water and ice film thickness needs to be clarified. In this study, different water and ice film thicknesses were prepared on the sensor element surface first. Then, the resonance frequency of the sensor with different film thicknesses of water and ice was recorded, as shown in Figure 2.

Figure 2 shows that the initial lg*f* value of the sensor was 3.5 (when the sensor surface was air), which was the turning point to distinguish whether the material on the sensor surface was water or ice. Resonance frequency was linearly correlated with the amount of water or ice covering the sensor. The denary logarithm of resonance frequency *f* was less than 3.5 when water was present on the sensor surface. As the water film thickness increased, the resonance frequency decreased because when the water volume increased, the electronic ceramic material’s equivalent stiffness was nearly unchanged, whereas the equivalent mass increased. When the sensor surface was covered with ice, the denary logarithm value of resonance frequency *f* exceeded 3.5. The resonant frequency increased with increasing ice thickness because the equivalent mass and stiffness of the electronic ceramic material increased, and the latter dominated the change in resonance frequency. According to this information, a sensor using piezoelectric technology can measure the thickness of ice or water to accurately acquire pavement surface information.

2. Sensor Structure

**Figure 3:**
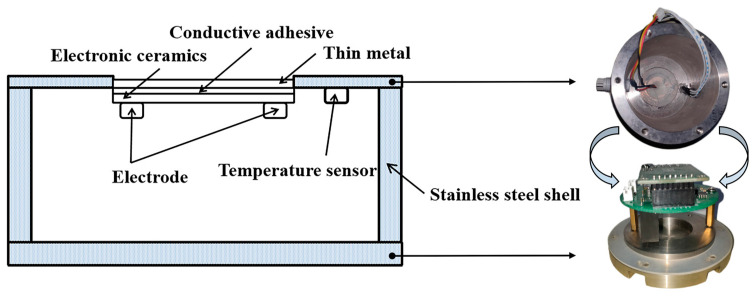
Schematic diagram of sensor structure.

The sensor in this study is composed of a shell, a base, an ice sensing element, a temperature sensor, a signal generator, and a microcomputer, as illustrated in Figure 3. The shell of the sensor was made of stainless steel to resist corrosion and loads, and the protection rank could reach IP68. For easy installation, a 74 mm diameter shell that was slightly smaller than the coring machine was used, and the height of the shell was 62 mm. By comprehensively considering the efficacy and size of the sensor, the AD9834 signal generator and STC12C5A60S2 microcontroller were selected to generate, collect, and transmit the electrical signals of the sensor. The signal generator and microcontroller was provided by Shenzhen Yatai Yingke Electronics Co., Ltd, Guangdong, China. Approximately 3000 RMB are required to produce a sensor with this configuration. This low-priced sensor is suitable for overlay applications in countries such as China, which has a vast network of roads.

#### 2.1.2. Sensor Operating Characteristics

1. Sensor Calibration

The developed piezoelectric sensor is expected to maintain good working characteristics within the operation temperature range. Therefore, different ice thicknesses were prepared at different temperatures to calibrate the sensor and examine its measurement accuracy. The accuracy of the sensor was calculated as Equations (2) and (3). The output value of the sensor was automatically calculated by complying with the relationship in Figure 2. The actual thickness of the ice or water film was calculated as the ratio of its volume to the surface area of the sensor.

(2) $$\varepsilon =\frac{\left|{Thickness}_{A}-{Thickness}_{O}\right|}{{Thickness}_{A}}\times 100\%$$

(3) $${Thickness}_{A}=\frac{{Volume}_{water/ice}}{Sensing\;element\;surface\;area}$$

where *Thickness_A_* denotes the actual thickness of ice or water film, mm; *Thickness_O_* is the output value of the sensor, mm.

**Figure 4:**
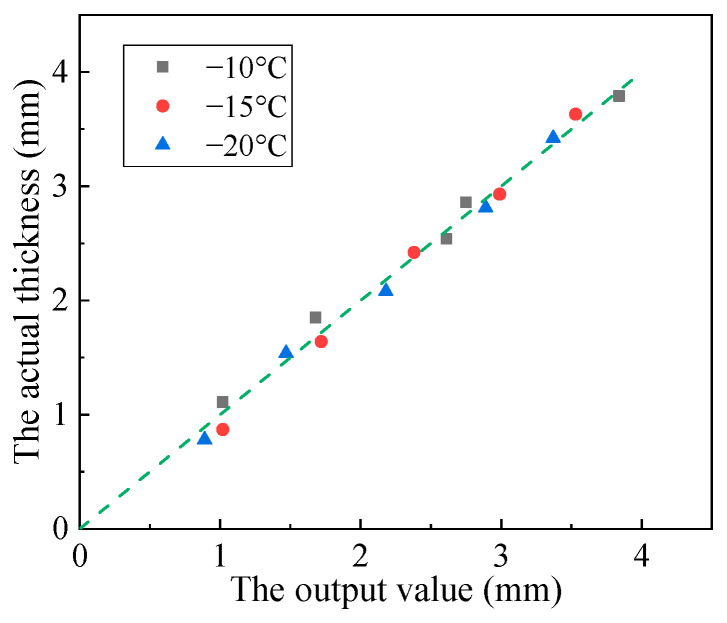
Accuracy of the sensor at different temperatures.

As shown in Figure 4, all data points were concentrated near the standard line. The deviation between the output and actual values was small. The average maximum error of the output value was less than 0.08 mm, which was only 2% of the measurement range. The accuracy of the sensor at −10, −15, and −20 °C was 4%, 3.7%, and 3.5%, respectively. This result indicates that the sensor can work in harsh environments with good accuracy.

2. Sensor Installation

**Figure 5:**
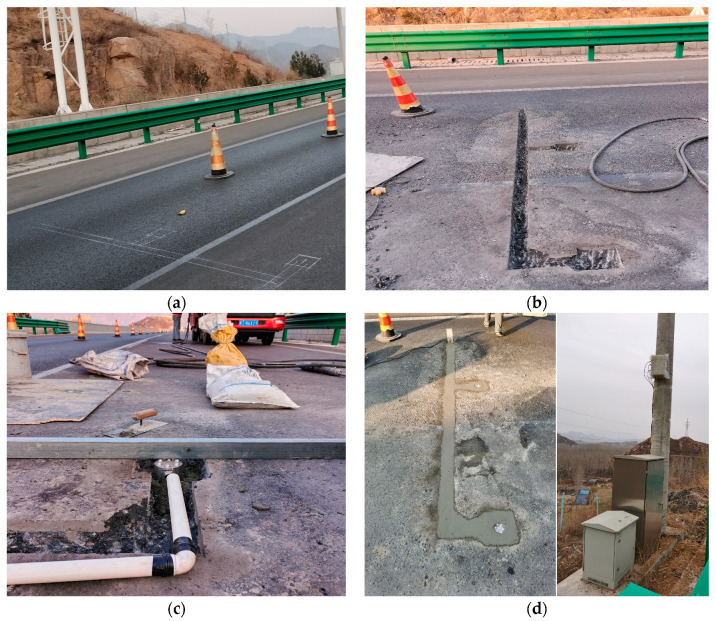
Installation of sensors in Laiqu Expressway in Hebei Province, China: (**a**) preparation of safety work; (**b**) grooving of pavement; (**c**) installation of the sensor; (**d**) rehabilitation of pavement and establishment of data transmission pathways.

In this study, two sensors were installed on an expressway surface to verify the long-term service performance of the piezoelectric sensors. The installation site was located in Laiqu Expressway in Hebei Province, China. The installation process included the preparation of safety work, the installation of the sensors, and the establishment of data transmission pathways, as shown in Figure 5. During installation, the sensing element was kept flush with the pavement surface. Data transmission was achieved using a data transmission box. The data transmission box was mounted on the roadside, connected to the sensors, and powered by 220 V of AC power. The box could quickly transmit monitoring data to the host computer via a wireless network with a delay of less than 0.1 s, thus enabling real-time detection of pavement icing.

**Figure 6:**
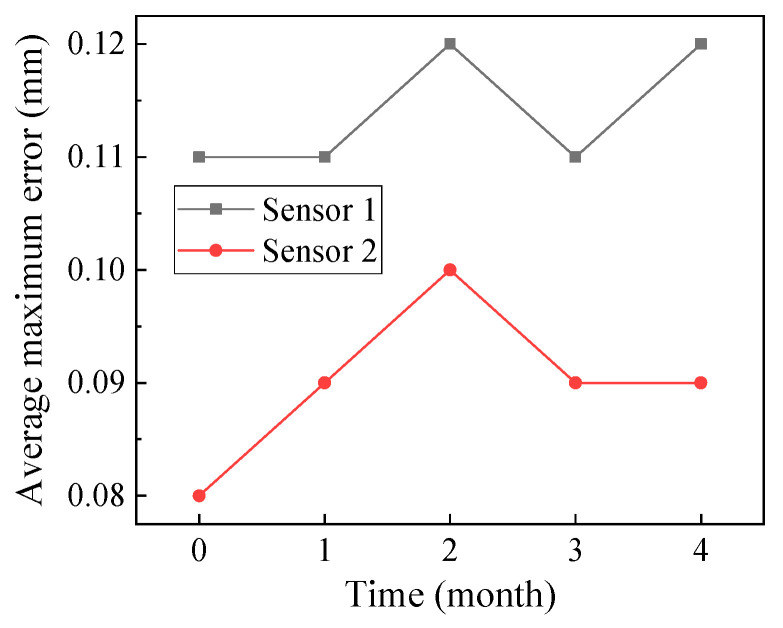
Average maximum error of the sensors in service.

**Figure 7:**
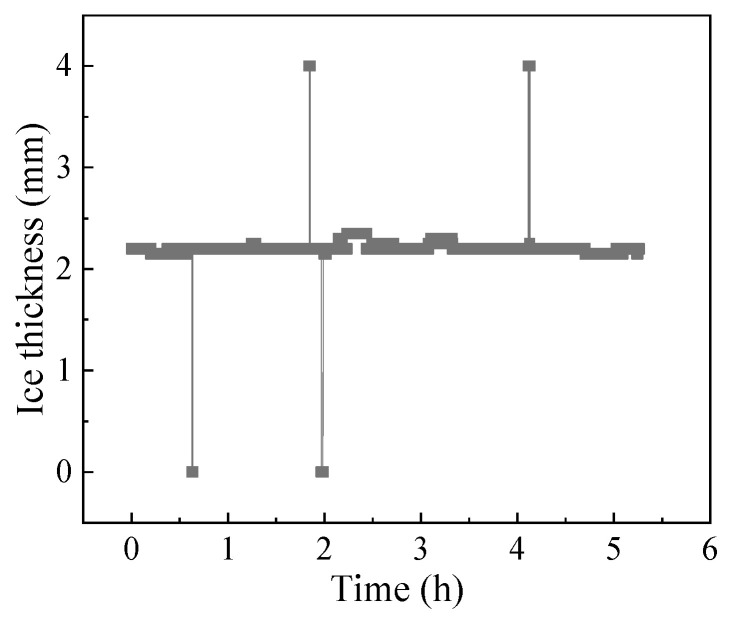
Examples of partial sensor monitoring data.

After four months of operation, the sensors were consistently in good condition and had an average maximum error of less than 0.15 mm, as shown in Figure 6. The sensors consistently demonstrated good accuracy and reliability when subjected to vehicle loads, temperature loads, and harsh natural environments in these days. Abnormal data were obtained during the monitoring process due to the electromagnetic influence. The abnormal data had a short duration and a considerable difference from the normal value. Some data are shown in Figure 7. Only four abnormal data points out of 5500 data points were obtained, and the probability of an abnormal data appearance was less than 1‰. Thus, the abnormal data could be easily identified and did not affect the actual monitoring effect. In summary, the proposed piezoelectric sensor exhibited high precision and good durability in pavement surface status monitoring and could provide effective information collection support for pavement icing early warnings.

### 2.2. Method

The effects of different factors (pavement temperature, wind speed, and water depth) on ice formation on the pavement surface were investigated from the outside. Icing time is the time when the water is completely frozen and can be obtained through sensor sensing. The resonant frequency of the sensor changes continuously as the water freezes. The water is considered completely frozen when the resonant frequency no longer changes. Laboratory tests were performed to quantify the influence of various factors on icing time. Then, orthogonal design-based tests were conducted to determine the importance of the factors to icing time. Notably, the experimental data here can be used as a database for pavement icing early warning models.

**Figure 8:**
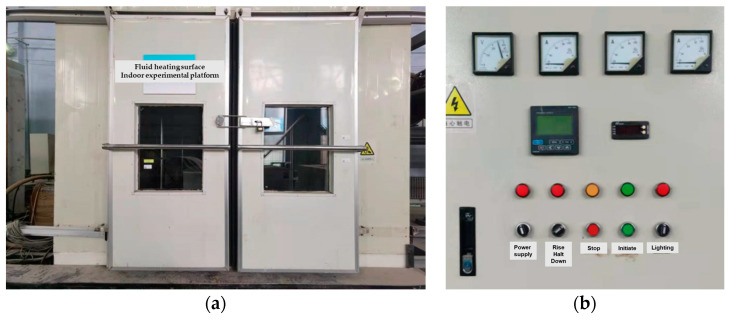
The climatic chamber: (**a**) chamber appearance; (**b**) temperature control system.

**Figure 9:**
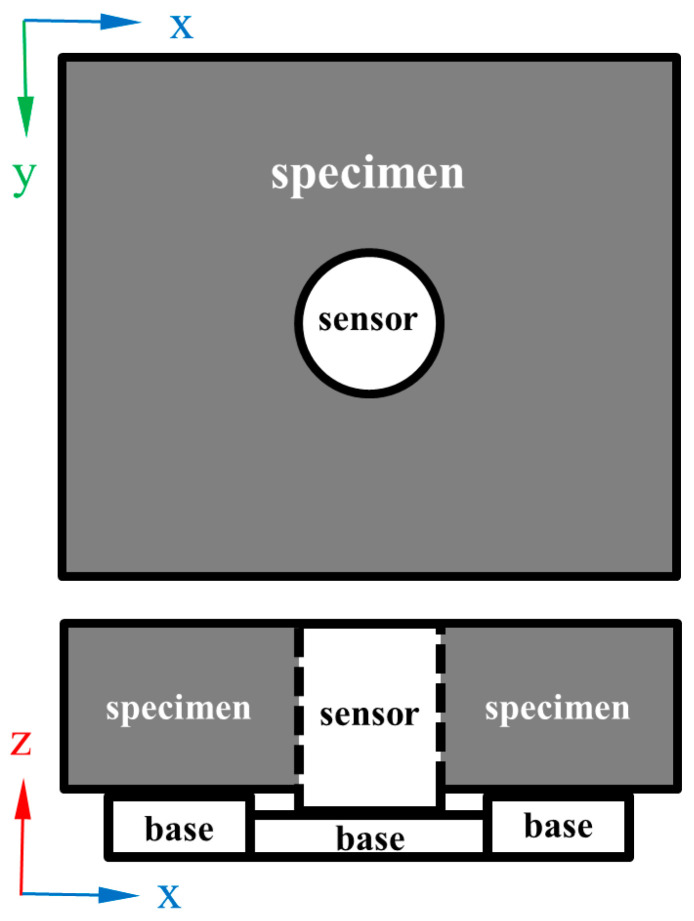
The sensor and the specimen.

The laboratory test equipment included a climatic chamber, a variable-speed fan, an ice sensor, and an AC-13 specimen whose length, width, and height were 300, 300, and 50 mm, respectively. The laboratory tests were performed in a climatic chamber with a length, width, and height of 3 m, as shown in Figure 8. The climatic chamber was assembled by the Harbin Institute of Technology Cold Region Road Research team. The environmental conditions included temperature, wind speed, and water depth. The temperature control accuracy of the climatic chamber was 0.1 °C, the wind speed was controlled by a variable-speed fan, and the water depth was prefabricated by quantitative weighing. The AC-13 asphalt mixture specimen was prepared using matrix 70 # asphalt and basalt aggregate. The parameters of the asphalt and aggregate met standard requirements. As shown in Figure 9, the sensor was embedded in the center of the specimen. A base was placed under the sensor and specimen to ensure that their surfaces were flush. The specimen and sensor were kept in the test environment for over an hour before testing, and water was placed at a temperature of 0 °C.

The conditions of the experimental environment were determined based on the local climate. The climate data of Hebei Province obtained over the past three years show that the average daily minimum temperature in winter is about −8 °C, the average annual wind speed is less than 5 m/s, and the average precipitation is less than 4 mm. In consideration of these climatic conditions, the experimental temperatures were set to −2, −4, −6, and −8 °C. The wind speeds were set to 1, 2, 3, and 4 m/s, and the water depths were set to 1, 1.5, 2.5, and 3.5 mm. The equal-level orthogonal L16 (4 × 4) test design was applied to the four factors and levels, and a null column was added between the factors to estimate the random error. The three factors and the null column were classified as A, B, C, and D columns, respectively. The design scheme is given in Table 1.

## 3. Results and Discussion

Temperatures in Hebei Province exceed 0 °C during the day and are below 0 °C at night. Snowfall melts during the day and freezes to ice at night due to the temperature difference between day and night. The low-temperature conditions at night and the residual precipitation on the pavement surface are the key factors for pavement ice formation in Hebei Province. In addition, the presence of wind can accelerate the icing process. Therefore, pavement temperature, wind speed, and water depth were selected as experimental variables, and their specific effects on icing time were elucidated. The experimental data served as a basis for the nondestructive detection of pavement icing.

### 3.1. The Effect of Wind Speed on Pavement Icing

**Figure 10:**
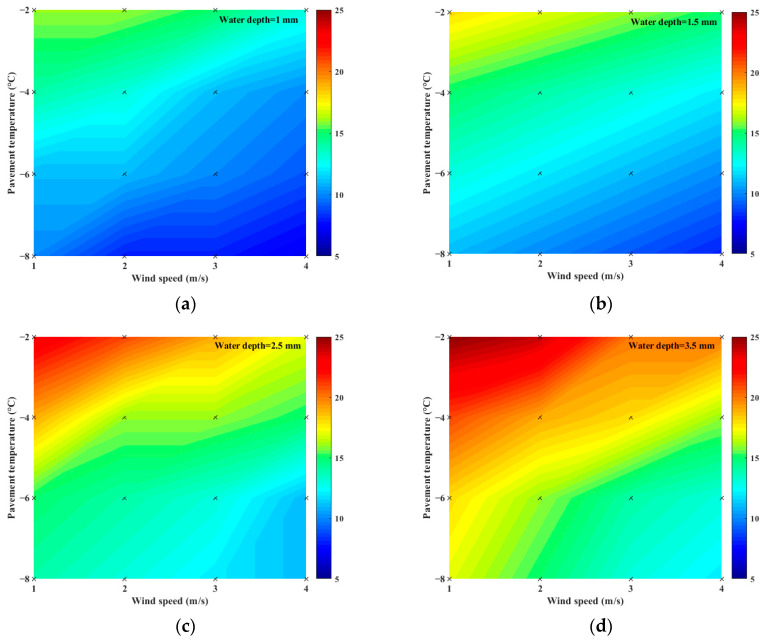
Effects of wind speed on icing time: (**a**) water depth = 1 mm; (**b**) water depth = 1.5 mm; (**c**) water depth = 2.5 mm; (**d**) water depth = 3.5 mm.

The presence of wind may accelerate energy dissipation and thus shorten the icing time. To analyze the effect of wind speed on icing time, icing time was tested at wind speeds of 1, 2, 3, and 4 m/s while maintaining a constant pavement temperature and water depth. Figure 10 depicts the relationship between icing time and wind speed. The color in the figure represents the icing time. As shown in the legend, the closer the color is to red, the longer the time is; the closer the color is to blue, the shorter the time is.

Under a certain pavement temperature and water depth, icing time increased with the decrease in wind speed. The shortening rate of icing time changed from 17% to 30% as the wind speed increased from 1 m/s to 4 m/s. This result proves that wind speed positively influenced icing time, but the degree of influence was small. The temperature of the water was close to that of the wind, and the internal energy of water could not easily exchange heat with the wind, causing a minor influence of wind speed on the icing time.

### 3.2. The Effect of Water Depth on Pavement Icing

**Figure 11:**
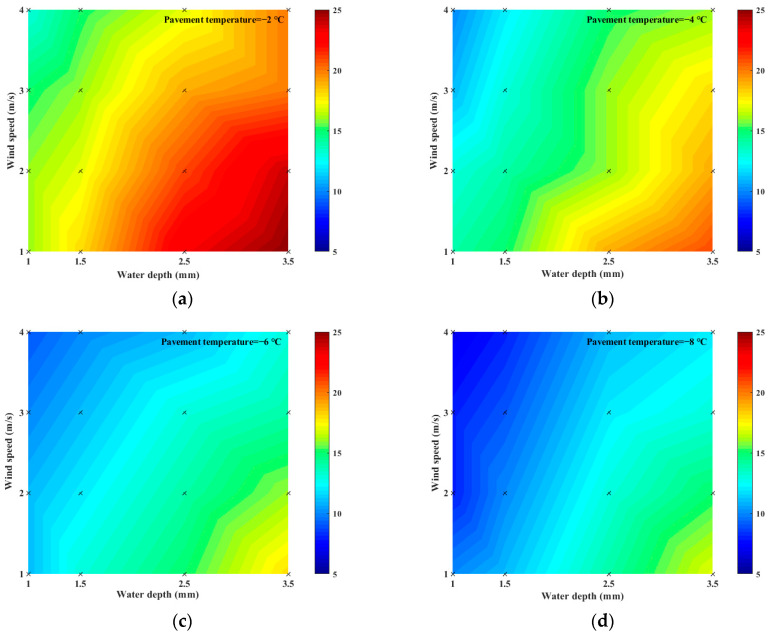
Effects of water depth on icing time: (**a**) pavement temperature = −2 °C; (**b**) pavement temperature = −4 °C; (**c**) pavement temperature = −6 °C; (**d**) pavement temperature = −8 °C.

In this section, it is assumed that the depths of the pavement surface structures are equal at all locations, and the amount of water on the pavement surface is quantified using the “water depth” (the value is equal to the water volume divided by the pavement area). To analyze the effect of water depth on the icing time, the experiment was conducted at water depths of 1, 1.5, 2.5, and 3.5 mm while keeping the temperature and wind speed constant. The results are presented in Figure 11.

When the pavement temperature and the wind speed remained unchanged, the icing time increased with the increase in the water depth. The shortening rate of the icing time changed from 25% to 47% as the water depth reduced from 3.5 mm to 1 mm. This result indicates that water depth negatively influenced icing time. Given the same contact area between water and other substances, the more water on the pavement surface, the slower the energy exchange between water and other substances, and the longer the icing time.

### 3.3. The Effect of Pavement Temperature on Pavement Icing

**Figure 12:**
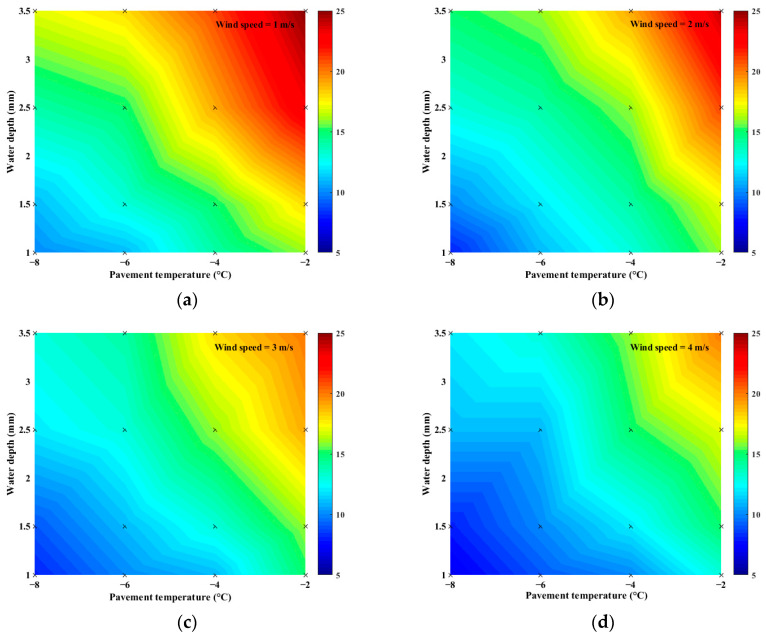
Effects of pavement temperature on icing time: (**a**) wind speed = 1 m/s; (**b**) wind speed = 2 m/s; (**c**) wind speed = 3 m/s; (**d**) wind speed = 4 m/s.

The pavement is in direct contact with water, and the pavement temperature will have some effect on the icing rate. To analyze the effect of pavement temperature on icing time, the experiment was conducted at pavement temperatures of −2, −4, −6, and −8 °C while keeping the water depth and wind speed constant. The results are presented in Figure 12.

For a given wind speed and water depth, the icing time increased with the decrease in the pavement temperature. The shortening rate of icing time varied from 32% to 50% and the pavement temperature decreased from –2 °C to –8 °C. The result indicates that the pavement temperature positively influenced the icing time. The effect of the pavement temperature on icing time is greater than the wind speed and water depth, which is identical to the conclusion of the orthogonal test above. The pavement temperature was lower than that of water, which made it convenient for water to release heat and freeze. Lower pavement temperatures can accelerate the energy exchange between water and pavement, resulting in faster condensation nucleation and crystallization of water.

In general, the essence of pavement surface icing is that water loses heat, resulting in a decrease in water molecular kinetic energy, condensation, nucleation, and crystallization. According to the above test results, the icing time increases with the increase in pavement temperature, the slowing of wind speed,* and the thickening of water depth. The maximum icing time is more than 25 min under the conditions of −2 °C pavement temperature, 1 m/s wind speed, and 3.5 mm water depth. And the minimum icing time is less than 8 min when the temperature is −8 °C, the wind speed is 4 m/s, and the water depth is 3.5 mm.

### 3.4. Significance Analysis of Variables

An equal-level orthogonal L16 (4 × 4) test was performed to compare the significance of the effects of pavement temperature, wind speed, and water depth on pavement icing. The variables were statistically ranked in terms of the degree of influence they had on icing time. The design and results of the experiment are presented in Table 2.

An orthogonal test usually uses ranges to distinguish primary and secondary factors. As indicated by the range analysis in Table 2, the ranges of the pavement temperature, wind speed, water depth, and the null column reached 29, 15, 25, and 5. The pavement temperature was the main factor that affected the icing time of pavements, followed by water depth and wind speed. In addition, Table 2 shows that the three factors with the highest levels were K1, K1, and K4, and A1B1C4 was the longest test condition of icing time. Subsequently, to analyze the significance of factors, the variance of the test results was calculated to conduct an F-test, as shown in Table 3.

According to Table 3, the F values of pavement temperature, wind speed, and water depth were 33.814, 9, and 25, respectively. All F values were greater than the F critical value (F (3,3,0.9) = 5.36), indicating that the three factors significantly influenced icing time. In addition, the smaller the *p* value was, the more significant the result was. The *p* values of three factors were small, indicating that these factors significantly influenced icing time.

Overall, the pavement temperature, wind speed, and water depth had significant effects on icing time, with the effects of pavement temperature and wind speed being positive and the effects of water depth being negative. Pavement temperature had the greatest effect on icing time (up to 50%), followed by water depth; the effect of wind speed was the smallest (as small as 17%). However, the effects of the three factors on icing time were nonlinear, and the coupling between them was difficult to explain by a unified empirical formula.

## 4. Early Warning Model

The future of road engineering is nondestructive road facility identification and warning, which is made possible by quick statistical analysis and potent machine learning techniques [29,30,31]. Accurate road icing warnings based on meteorological data are required to achieve nondestructive detection of road ice. Given that the factors contributing to pavement icing are interrelated and complex, pavement icing early warning should comprehensively consider the nonlinear relationship of multiple variables. An artificial neural network (ANN) is an adaptive multilayer network that is particularly suitable for solving this problem due to its complex internal mechanisms, which have been proven by mathematical theory.

**Figure 13:**
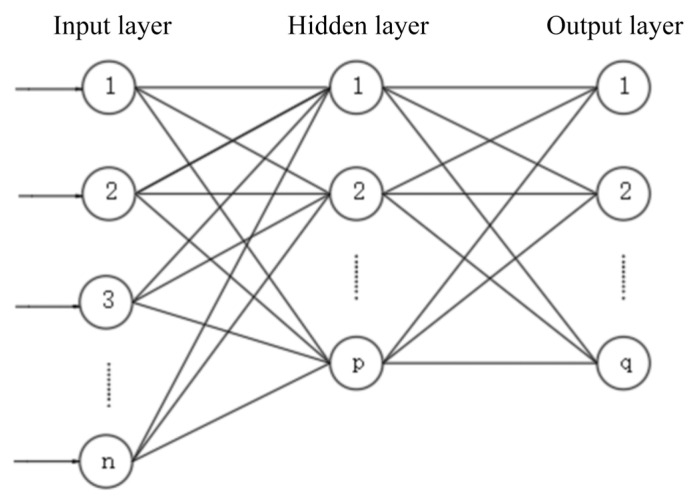
The structure of single hidden-layer neural network.

The single hidden-layer backpropagation (BP) training algorithm of ANN was selected to predict the pavement icing time in this study, as shown in Figure 13.

S-shaped neurons were used in the hidden layer, and linear neurons were used in the output layer. The transfer function is a (0,1) S-shaped function, which is shown in Equation (4).

(4) $$f(x)=\frac{1}{1+e^{-x}}$$

The operation of the network includes a series of steps: network initialization, importing sample data, the output calculation of the network, the partial derivatives calculation of the error function, adaptation of the connection weight, and the calculation of the sample error. The final sample error formula is shown in Equation (5). The process is terminated if the error satisfies the predetermined accuracy standards or has been trained a maximum number of times; otherwise, network training is repeated.

(5) $$E=\frac{1}{2m}\sum_{k=1}^{m}\sum_{s=1}^{q}{\left(t_{s}\left(k\right)-yo_{s}\left(k\right)\right)}^{2}$$

where *m* denotes the number of samples; *t*(*k*) is the desired output; *yo*(*k*) is the output of network.

The results in Section 4 show that pavement temperature, water depth, and wind speed had strong correlations with icing time. Thus, the three meteorological parameters were adopted as input parameters, and icing time was employed as an output parameter. In addition, the number of hidden units and the learning rate, which have a significant effect on calculation speed and result accuracy, served as vital parameters of the BP neural network. The prediction accuracy of the model with different numbers of hidden units and learning rates was determined to reduce the prediction error. The database consisted of 64 groups of test data from the previous laboratory experiment, as shown in Table 4. The experimental temperatures were set to −2, −4, −6, and −8 °C. The wind speeds were set to 1, 2, 3, and 4 m/s, and the water depths were set to 1, 1.5, 2.5, and 3.5 mm. Icing times were recorded for 64 combinations of the above conditions. The experimental data were randomly arranged to ensure the randomness of the model input data. Fifty-one groups (80%) of randomly selected laboratory test data were used to train the prediction model, and the remaining test data (20% of randomly selected laboratory test data) served as a testing set to test the prediction accuracy of the model.

### 4.1. Number of Hidden Units

**Figure 14:**
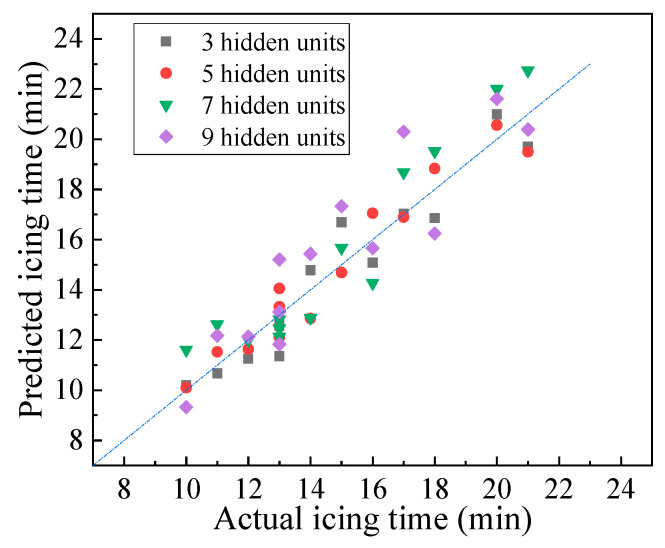
Deviation of predicted values of models with different number of neurons.

A hidden unit is a fundamental computing unit in a neural network’s hidden layer that receives and processes data and corrects the weight coefficients. The hidden layer of a neural network contains several hidden units, and the number of hidden units can be used to determine the goodness of fit of the neural network. A model with a small number of hidden units cannot easily acquire sufficient information from the learning set, which may cause model underfitting. Meanwhile, a training network with too many hidden units may cause model overfitting and insufficient generalization ability. In this study, the numbers of hidden units were 3, 5, 7, and 9 when the BP neural network prediction model was implemented on the training set. The prediction accuracy of the model was analyzed based on the testing set. The results are shown in Figure 14 and Table 5.

According to Figure 14 and Table 5, accuracy initially increased, then decreased with the increase in the number of hidden units. When the number of hidden units was 5, the average, maximum, and root mean square errors of the model were 0.67, 1.50, and 0.79 min, respectively, which are the minimum values for different models. This result indicates that the predicted value of the current model is the closest to the real value. The Pearson test showed that the model’s goodness of fit was the highest. Thus, the optimal number of hidden units for the single-layer neural network model was determined to be 5. The neural network with five hidden units can predict pavement icing time efficiently.

### 4.2. Learning Rate

**Figure 15:**
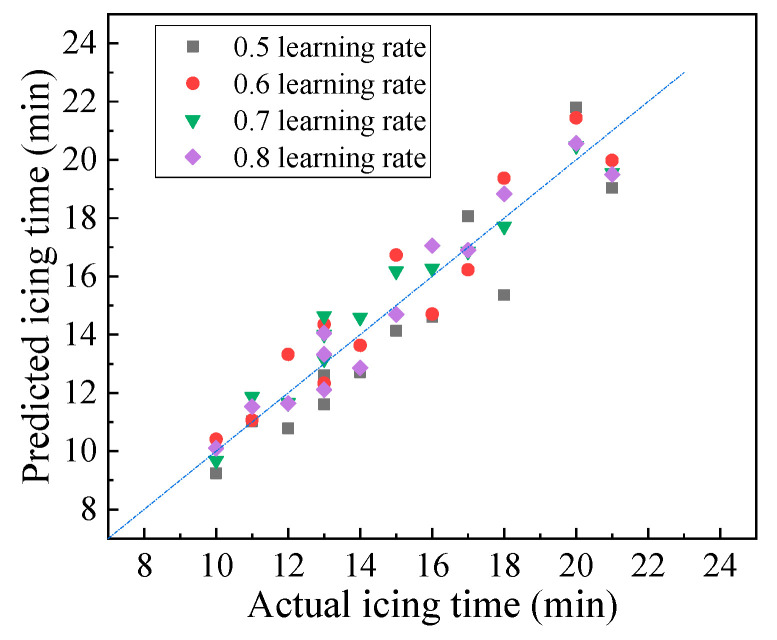
Deviation of predicted values of models with different learning rates.

BP neural network models are typically trained with the gradient descent method, and the learning rate is related to the distance that determines how far the weights move in the gradient direction. The learning rate determines whether and when the objective function converges. Low learning rates decrease the convergence speed of the model. However, if the learning rate is too high, network shock or even non-convergence may occur. The BP neural network prediction model with learning rates of 0.5, 0.6, 0.7, and 0.8 was trained in this work. Then, the prediction accuracy of the model was analyzed based on the testing set. The results are shown in Figure 15 and Table 6.

Figure 15 and Table 6 show that with the increase in the learning rate, accuracy initially increased then decreased, and this pattern was consistent with the change law of the number of hidden units. When the learning rate was 0.7, the average, maximum, and root mean square errors of the model were the smallest, and the Pearson correlation coefficient was the largest. The optimal learning rate of the neural network model was determined to be 0.7. Therefore, the neural network with five hidden units and a learning rate of 0.7 predicts the pavement icing time the best, and it may be valuable for pavement icing warning work.

### 4.3. The Validation of The Model

**Figure 16:**
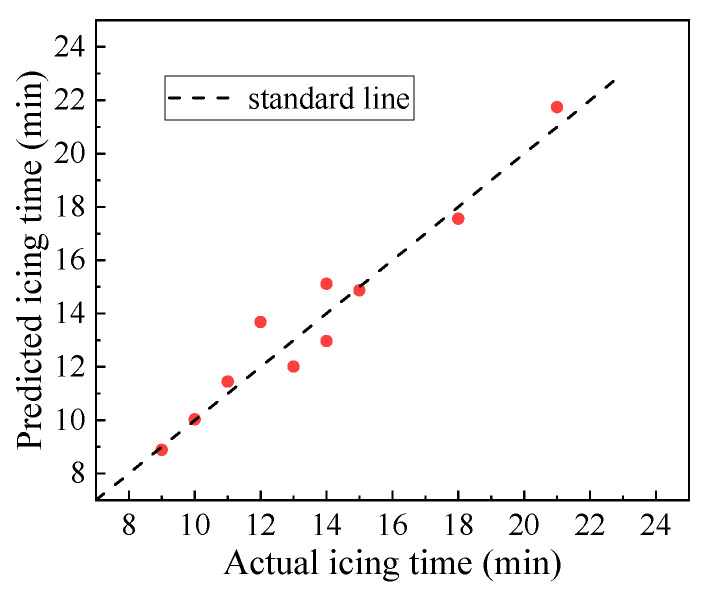
Comparison between the actual icing time and the icing time predicted by B.P. neural network model.

The accuracy and validity of the model were assessed using the validation dataset. The three meteorological conditions, namely, temperature, wind speed, and water depth, were inputted into the BP neural network model to obtain the predicted icing time. The prediction results of the model are shown in Figure 16 and Table 7.

As indicated in Figure 16 and Table 7, the predicted values of the icing time obtained from the BP neural network model had small errors, with the mean, maximum, and root mean square errors being 0.71, 1.83, and 0.83 min, respectively. The Pearson correlation coefficient between the predicted and measured values was 0.986, indicating a good correlation between the two. In addition, the model’s prediction accuracy reached 90.7, which indicates an accurate prediction of the pavement icing time.

To sum up, the single-layer BP neural network model that uses pavement temperature, water depth, and wind speed as the input parameters successfully predicted the pavement icing time. The model with a learning rate of 0.7 and five hidden units had the best prediction effect on pavement icing, and its accuracy reached 91.7%. The model can be used for pavement pre-icing warning and can help prevent traffic accidents. Given that the BP neural network model is an adaptive learning network, the parameters of this model are applicable to areas at the same latitude and longitude as Hebei Province.

## 5. Conclusions

In this study, an icing nondestructive detection and early warning system for asphalt pavements was established. First, the principle and characteristics of the sensors were introduced, and the performance of the sensors was investigated. Second, the icing time under various operating situations was studied, and a highly accurate, self-adaptive early warning model for pavement icing was developed. The main research results are as follows:

- The piezoelectric sensors were designed to quantitatively indicate the substance (i.e., air, water, or ice) covering the sensors’ surfaces in accordance with the function of substance thickness and resonance frequency. lg*f* showed a linear relationship with ice and water thickness; it decreased with the increase in water thickness and increased with the increase in ice thickness. The piezoelectric sensors exhibited the advantages of low cost, strong anti-interference ability, high measurement accuracy, and long survival time and are thus suitable for pavement surface condition monitoring.
- In the experiments, the effects of pavement temperature, wind speed, and water depth on pavement icing time were studied. The results showed that icing time increased with the increase in pavement temperature, the slowing of wind speed, and the thickening of water depth. Pavement temperature was the critical factor in determining icing time, and it could reduce icing time by 50% under normal winter conditions in Hebei Province. With the action of the three factors, the longest time for pavement icing exceeded 25 min, and the shortest time was only 8 min.
- On the basis of the influence law of wind speed, water depth, and pavement temperature, a warning for pavement icing was provided by the BP neural network model. The BP neural network model with a learning rate of 0.7 and five hidden units exhibited optimal prediction performance in pavement icing early warning, and its prediction accuracy was as high as 91.7%.

In this study, the early warning system was adopted to warn of ice caused by freeze–thaw of precipitation and black ice attributed to air humidity in central and southern China, without considering the water type (contamination) and the snowfall in northern areas. The influence of other contaminations, such as sand and lubricating oil, on the detection effects was also not taken into account. In addition, the usefulness of the early warning system needs to be further improved, and more powerful statistical and machine learning methods should be used. The focus of the follow-up studies will be centered on the above aspects, depending on the climatic conditions in cold regions. Thus, the general applicability of the pavement icing early warning system can be improved to better support the safe operation of expressway traffic.

## Figures and Tables

**Table 1:** Factors and level of orthogonal test.

Level	Pavement Temperature (°C)	Wind Speed (m/s)	Water Depth (mm)	Null
1	−2	1	1	1
2	−4	2	1.5	2
3	−6	3	2.5	3
4	−8	4	3.5	4

**Table 2:** Factors and level of orthogonal test.

**Number**	1	2	3	4	5	6	7	8	9	10	11	12	13	14	15	16	K1	K2	K3	K4	Range
**Pavement temperature (°C)**	−2	−6	−4	−2	−6	−6	−4	−2	−8	−8	−4	−2	−8	−6	−8	−4	73	62	50	44	29
**Wind speed (m/s)**	4	2	1	2	4	3	3	1	4	3	2	3	1	1	2	4	64	61	55	49	15
**Water depth (mm)**	2.5	2.5	3.5	3.5	3.5	1	2.5	1	1	3.5	1	1.5	2.5	1.5	1.5	1.5	46	51	61	71	25
**Null**	3	1	3	4	2	3	4	1	4	1	2	2	2	4	3	1	55	56	58	60	5
**Icing time (min)**	17	14	21	24	13	10	16	16	7	13	13	16	14	13	10	12	-	-	-	-	-

**Table 3:** The variance analysis of orthogonal test.

Factor	Degree of Freedom	MS	F	*p*
Pavement temperature	3	41.562	33.814	0.008
Wind speed	3	11.062	9	0.052
Water depth	3	30.729	25	0.013
Error	3	1.229	-	-

**Table 4:** Data basis of the artificial neural network.

No.	Pavement Temperature (°C)	Wind Speed (m/s)	Water Depth (mm)	Icing Time (min)	No.	Pavement Temperature (°C)	Wind Speed (m/s)	Water Depth (mm)	Icing Time (min)
1	−6	4	1	9	33	−6	2	1.5	12
2	−4	2	3.5	19	34	−2	4	1	13
3	−6	3	3.5	14	35	−2	4	1.5	15
4	−6	1	1	11	36	−4	4	3.5	16
5	−6	3	1	10	37	−8	2	2.5	13
6	−8	4	3.5	12	38	−8	4	2.5	11
7	−8	3	1	8	39	−2	1	2.5	23
8	−2	3	2.5	19	40	−6	3	2.5	13
9	−6	1	1.5	13	41	−4	4	1	10
10	−2	4	3.5	20	42	−4	2	2.5	16
11	−8	1	3.5	17	43	−6	4	2.5	11
12	−2	2	1	16	44	−6	1	3.5	18
13	−6	3	1.5	11	45	−2	1	1.5	18
14	−8	1	2.5	14	46	−4	4	2.5	15
15	−8	4	1.5	8	47	−6	2	2.5	14
16	−8	2	1.5	10	48	−8	2	1	8
17	−4	1	2.5	19	49	−8	3	3.5	13
18	−8	3	1.5	9	50	−2	3	1.5	16
19	−6	2	3.5	16	51	−2	1	3.5	25
20	−8	1	1	10	52	−4	3	2.5	16
21	−4	3	1	11	53	−4	4	1.5	12
22	−2	1	1	16	54	−6	1	2.5	15
23	−8	4	1	7	55	−4	3	3.5	18
24	−8	2	3.5	15	56	−8	1	1.5	11
25	−4	1	3.5	21	57	−6	4	3.5	13
26	−4	1	1	14	58	−2	3	3.5	20
27	−2	2	1.5	17	59	−2	4	2.5	17
28	−2	2	3.5	24	60	−4	2	1.5	14

**Table 5:** Model prediction under different number of neurons.

Number of Hidden Units	Average Error (min)	Maximum Error (min)	Accuracy (%)	Root Mean Square Error (min)	Pearson Correlation Coefficient
3	0.81	1.69	90.6	0.96	0.961
5	0.67	1.50	91.7	0.79	0.970
7	1.18	2.00	88.9	1.33	0.953
9	1.30	3.31	81.6	1.59	0.913

**Table 6:** Model prediction under different learning rates.

Learning Rate	Average Error (min)	Maximum Error (min)	Accuracy (%)	Root Mean Square Error (min)	Pearson Correlation Coefficient
0.5	1.19	2.64	85.3	1.36	0.921
0.6	0.96	1.73	90.4	1.08	0.943
0.7	0.67	1.50	91.7	0.79	0.970
0.8	0.67	1.64	90.9	0.83	0.963

**Table 7:** Prediction results of the B.P. neural network model.

Average Error (min)	Maximum Error (min)	Accuracy (%)	Root Mean Square Error (min)	Pearson Correlation Coefficient
0.71	1.68	90.7	0.83	0.986

## Data Availability

Not applicable.
